# Early Fixation of Cobalt-Chromium Based Alloy Surgical Implants to Bone Using a Tissue-engineering Approach

**DOI:** 10.3390/ijms13055528

**Published:** 2012-05-09

**Authors:** Munehiro Ogawa, Yasuaki Tohma, Hajime Ohgushi, Yoshinori Takakura, Yasuhito Tanaka

**Affiliations:** 1 Department of Orthopedic Surgery, Nara Medical University, 840 Shijyo-cho, Kashihara, Nara 634-8522, Japan; E-Mails: mogawa@naramed-u.ac.jp (M.O.); tohma@wnara.hosp.go.jp (Yasua.T.); ashitakakura@leto.eonet.ne.jp (Yo.T.); yatanaka@naramed-u.ac.jp (Yasuh.T.); 2 Department of Orthopedic Surgery, National Hospital Organization Nara Medical Center, Nara 630-8053, Japan; 3 Health Research Institute, National Institute of Advanced Industrial Science and Technology (AIST), Amagasaki, Hyogo 661-0974, Japan

**Keywords:** implant-bone interface, cobalt chromium alloy, marrow mesenchymal cell, osteogenesis, tissue engineering

## Abstract

To establish the methods of demonstrating early fixation of metal implants to bone, one side of a Cobalt-Chromium (CoCr) based alloy implant surface was seeded with rabbit marrow mesenchymal cells and the other side was left unseeded. The mesenchymal cells were further cultured in the presence of ascorbic acid, β-glycerophosphate and dexamethasone, resulting in the appearance of osteoblasts and bone matrix on the implant surface. Thus, we succeeded in generating tissue-engineered bone on one side of the CoCr implant. The CoCr implants were then implanted in rabbit bone defects. Three weeks after the implantation, evaluations of mechanical test, undecalcified histological section and electron microscope analysis were performed. Histological and electron microscope images of the tissue engineered surface exhibited abundant new bone formation. However, newly formed bone tissue was difficult to detect on the side without cell seeding. In the mechanical test, the mean values of pull-out forces were 77.15 N and 44.94 N for the tissue-engineered and non-cell-seeded surfaces, respectively. These findings indicate early bone fixation of the tissue-engineered CoCr surface just three weeks after implantation.

## 1. Introduction

Initial implant fixation is critical for long term performance of prosthetic arthroplasty. Therefore, implants ability to provide early, stable, and osseous fixation is required to ensure success in clinical cases. Incomplete anchorage between the implants surface and bone might lead to aseptic loosening and subsequent failure in the prostheses [1]. Cobalt chromium (CoCr) based alloys have been widely used for prosthetic arthroplasty. Their mechanical properties seem to be suitable material for the purpose of total hip and knee joint arthroplasty [2,3]. Recent reports, however, have raised some questions concerning the osteogenic function of the CoCr alloy, which might cause loosening of arthroplasty using this alloy [4,5].

We have developed a novel method to solve the problem of loosening of alumina ceramics ankle arthroplasty using a tissue engineering approach [6]. Mesenchymal cells residing in bone marrow can differentiate into osteoblasts and undergo mineralization when they are cultured in the presence of ascorbic acid, β-glycerophosphate and dexamethasone [7–10]. On the basis of these findings, we have succeeded in fabricating a tissue engineered alumina ceramics implant with excellent osteogenic function, which improves bone-implant fixation. Specifically, the method involves culturing the mesenchymal cells on the alumina ceramics implant surface prior to implantation. The culture shows osteogenic differentiation of the cells; *i.e.*, appearance of osteoblasts which fabricate bone matrix on the alumina ceramics implant. Previously, we reported that tissue engineered alumina ceramics caused early bone ingrowth in the rabbit model. In brief, the osteoblast/bone matrix formed on alumina ceramics can show further *in vivo* osteogenic function resulting in a stable interface between the tissue engineered alumina ceramics surface and the host bone [11]. Moreover, we also reported successful clinical cases of the tissue engineered alumina ceramics ankle arthroplasty using mesenchymal cells derived from patient bone marrow. These cases showed stable interface between the tissue engineered ceramics surface and host bone even some years post operation [6].

In the present study, we focused on CoCr alloys and hypothesized that this tissue engineering approach could be suitable for not only alumina ceramics implant but also CoCr based alloy implant in order to solve the inherent problems of this alloy such as loosening. For this purpose, we conducted the following study in which CoCr alloy implants were loaded with cultured mesenchymal cells and implanted in rabbit bone. Three weeks after the implantation, mechanical as well as histological analyses were performed to demonstrate early fixation of the alloy to the bone.

## 2. Results and Discussion

### 2.1. *In Vitro* Experiment

**Figure 1:**
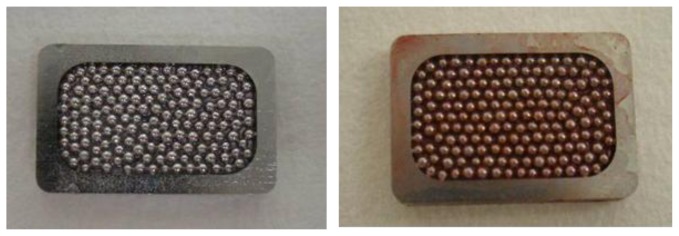
Alizarin red S stain of CoCr based alloy. Alizarin red S stain of the mesenchymal cells loaded (right) and non-cell loaded (left) surface of the alloy after *in vitro* osteogenic culture. Red color indicating bone mineral is only seen in the right figure.

We performed a tissue engineering approach for CoCr based alloy implant in order to solve problems of this alloy concerning the osteogenic property. The approach utilized marrow cells, which contain mesenchymal cells having osteogenic functions, and consisted of three steps: (1) Proliferation of mesenchymal cells from rabbit bone marrow by culture, (2) Osteogenic differentiation of the culture expanded cells resulting in the appearance of bone-forming osteoblasts together with bone matrix formation on the CoCr alloy and (3) Implantation of the the CoCr alloy in the same rabbit. To obtain the mesenchymal cells for step 1, we aspirated about 2 mL of rabbit bone marrow by needle. Therefore, the marrow harvest was performed in a minimally**-**invasive manner and the 2 mL marrow was enough to expand the number of mesenchymal cells for the experiments. During step 2, the culture mesenchymal cells differentiated into bone forming osteoblasts. The osteoblasts synthesize extracellular matrix in which bone mineral exist. The mineral can be stained with alizarin red S (Figure 1), and we conducted the stain to confirm bone matrix formation [9]. As seen in Figure 1, Alizarin red S was positive on the cell loaded surface. However, staining was negative on the non-cell loaded surface. The results confirmed that the surface of CoCr alloy was covered with osteoblasts and bone matrix and demonstrated the fabrication of tissue engineered CoCr alloy implants.

### 2.2. Mechanical Testing

**Figure 2:**
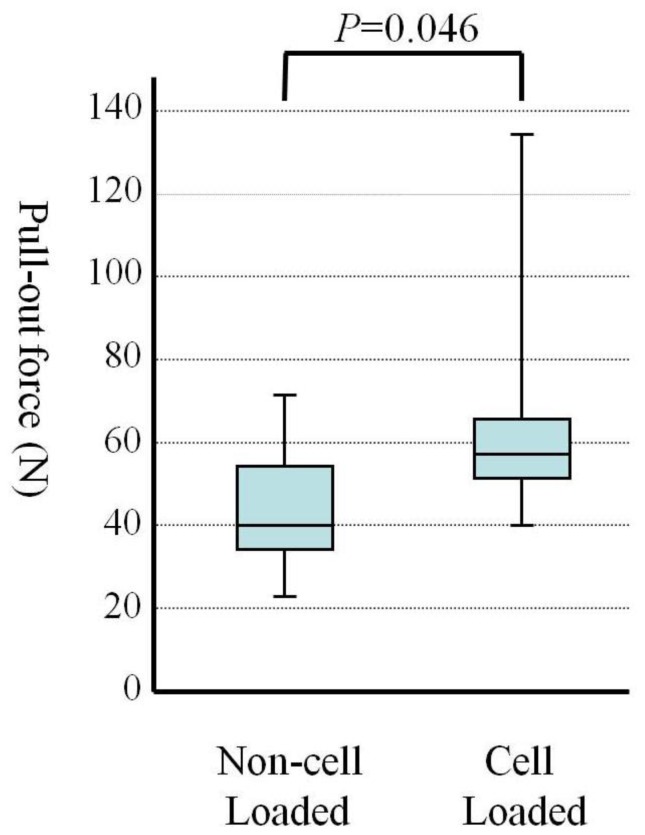
Pull-out forces of the implants after 3 weeks implantation (*n* = 6). The data are indicated by box plot. This plot is used to visually summarize and compare groups of data. The box plot uses the median, the approximate quartiles, and the lowest and highest data points to convey the level, spread, and symmetry of a distribution of data values. The parameters used in this figure are as follows: 50 percentiles as median, median is shown as a horizontal bar in the box.; 25 and 75 percentiles as a concentration indicator, 75 percentiles is shown as a top of the box and 25 percentiles is shown as a bottom of it; minimum and maximum data as a distribution range.

After the *in vitro* culture of mesenchymal cells on the CoCr alloy, we performed step 3. This consisted of *in vivo* implantation, to examine whether the tissue engineered CoCr implants exhibited the osteogenic function resulting in tight fixation of the implants to host bone. After creation of a bone defect in the rabbit tibia, we inserted the CoCr implants as described later in the methods section. The implantation was performed on the same rabbit from which we harvested the bone marrow. The pull-out test was performed on 6 tibias after 3 weeks implantation. In all cases, the implants detached from the non-cell loaded side. Then, the implant was again placed in the grip of the testing machine to evaluate pull-out force between the cell loaded implant surface and the bone on the opposite side. The mean values of pull-out force are shown in Figure 2. The pull-out force of the cell loaded side was greater than that of the non-cell loaded side. The mean values of pull-out forces were 77.15 N and 44.94 N for the cell loaded side and non-cell loaded side, respectively. The differences of the mean values of pull-out force between non-cell loaded side and cell loaded side were significant (*p* = 0.046) (Figure 2). After the pull-out test of non-cell loaded side, the bare surface of the CoCr implants was seen. Remnants of bone tissue were found after the detachment of cell loaded side.

### 2.3. Histological Examination and Electron Microscope Analysis

**Figure 3:**
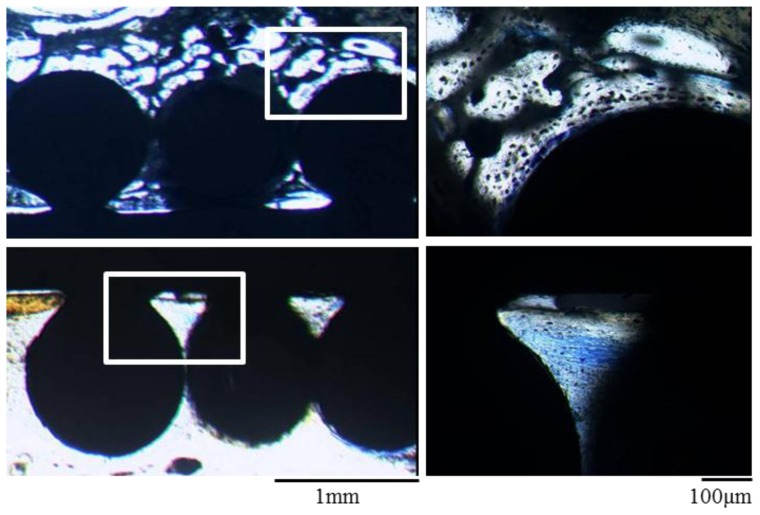
Toluidine blue-stained sections of the implants after 3 weeks. The cell loaded surface (upper images) shows extensive new bone formation into the spaces among the CoCr beads on the implant surface. The non-cell loaded surface (lower images) shows only connective tissue layers. Right figures are magnified images of the rectangular areas in the left figures.

Histological examination of the implants of the non-cell loaded surface showed a connective tissue layer at the interface between bone and the implant surface. However, there was no bone ingrowth into the spaces between the CoCr based alloy beads on the implant surface. On the other hand, the cell loaded surface showed new bone ingrowth into the spaces between the CoCr based alloy beads on the implant surface. Thus, extensive newly formed bone was detected in the tissue engineered implant surface and these histological findings verify the results of mechanical testing which demonstrate the tight fixation of the cell loaded implant surface and host bone (Figure 3).

**Figure 4:**
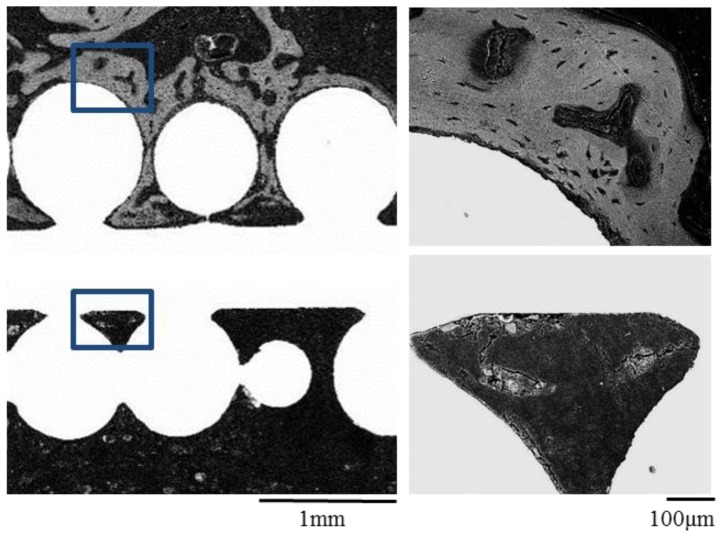
Electron microscope images of the implants after 3 weeks. As seen in Figure 6, abundant new bone formation is seen on the cell loaded surface (upper figures) but not on non-cell loaded surface (lower figures). Right figures are magnified images of the rectangular areas in the left figures.

Electron microscope analysis was consistent with the results of histological examination. Electron microscope images of the cell loaded surface exhibited abundant new bone formation. However, newly formed bone tissue was difficult to detect on the side of the non-cell loaded surface (Figure 4).

### 2.4. Discussion

Metallic biomaterials have a wide range of applications as prosthetic materials for joint arthroplasty in the orthopedic field. In recent years, the use of Titanium (Ti) based alloys as biomaterials have increased due to superior biocompatibility and corrosion resistance compared to other metallic alloys. The Ti alloys, however, have a disadvantage in wear resistance due to lower elastic modulus/hardness [12] and alumina ceramics are attractive as an implant material for the bearing surface of prosthetic arthroplasty due to their high abrasion resistance and hardness. Vickers hardness of Ti alloy is approximately 340 HV and that of alumina ceramics is approximately 2000 HV [13,14]. On the other hand, fracture toughness of the alumina ceramics (less than 4 MPa m^1/2^) is poor when compared to that of metals such as CoCr alloy (less than 100 MPa m^1/2^) [13,15] and fabrication of complex shapes of the ceramics is difficult. As to the Young’s modulus, the modulus of CoCr alloys (220–230 GPa) is about twice than that of Ti alloys (100–110 GPa) [16]. Thus, CoCr based alloys possess superior stiffness and toughness, furthermore, a recent method [17] significantly improved the mechanical property of CoCr alloys. Therefore, the alloys have been used as an alternative to Ti alloys and alumina ceramics in the orthopedics industry. However, several studies of CoCr alloy implantation have reported the possibility of risks to successful arthroplasty when using such alloys [4].

Soluble ions such as cobalt, which is a major component of CoCr alloy implants, are known to promote bone resorption [18] and inhibit proliferation/mineralization of bone marrow cells [19]. The metal ions also stimulate inflammatory cytokines and have a cytotoxic effect on cells surrounding the implants [4,20–24]. Thus, released metal ions from the CoCr implants might disturb local bone homeostasis at the bone-implant interface, leading to bone loss and thus resulting in aseptic loosening of the implant. If we control the bone homeostasis to promote bone formation around the implants, the bone loss could be prevented. One possible way is to supply osteogenic function to the implants prior to their implantation, because Co ions are reported to inhibit the osteogenic differentiation capability of marrow mesenchymal cells [19]. We thus culture-expanded the number of mesenchymal cells from bone marrow and then loaded the cells on the CoCr alloys. We further cultured the cells loaded CoCr alloys in the presence of ascorbic acid, β-glycerophosphate and dexamethasone and then implanted into rabbit bone defects.

As shown in Figure 1, positive staining for Alizarin red S on the CoCr alloy implants with mesenchymal cells loading indicated the appearance of osteoblasts and bone matrix formation on the alloy surface [25]. Thus, we succeeded in generating thin layer of tissue engineered bone on one side of the CoCr alloy. The alloys were implanted with due consideration given to the differences of the triangular cross-sectional geometry of the tibia as described later in the methods section. After 3 weeks, all the implants were analyzed. In mechanical testing, the non-cell loaded sides in all the implants detached first even though the tibia surface area facing the implants was larger. There was a significant difference between the pull-out force of the non-cell loaded surface and that of the cell loaded surface (Figure 2). Furthermore, histological and electron microscope images of the cell loaded surface exhibited abundant new bone formation (Figures 3,4). These findings suggest that the newly formed bone on the cell loaded CoCr surface interlocked the implants and, importantly, tight fixation could be obtained just 3 weeks after implantation. We reported that the tissue engineered bone on the ceramics surface well integrated to host bone [26] and also experienced good clinical cases using alumina ankle arthroplasty [6], therefore prolonged stable fixation between the CoCr surface and host bone could be expected.

While the use of poly(methyl methacrylate) (PMMA) bone cements may show good implant fixation, their disadvantages have been reported. They include toxicity of PMMA [27], decreased bone stock at the time of revision, difficulty in the treatment after infection around the implants and weakening of the fixation over time [28]. As a result, various authors have advocated cementless fixation, especially for young and active patients [29,30], and some retrospective studies have reported better results for cementless fixation compared to cemented [31,32]. The main disadvantage of the cementless fixation, however, is poor fixation in the early period after the implantation. To promote early fixation, rough surfaces of the implants were adopted. One type of surface is the metal-bead coated implant as used in the present study. However, bony ingrowth into the rough surface may take some months so the issue with the cementless fixation is how to obtain stable early fixation [33–35]. Therefore, the use of CoCr alloy implants is inappropriate for the purpose of cementless fixation because they impair the osteogenesis around their implants as discussed above. In this study, loading and culturing the cells on the CoCr implants prior to implantation achieved stable early bone fixation. These findings suggest that the tissue engineered CoCr implants might be used for cementless fixation in joint arthroplasty.

Coating the metal implants with the chemically synthetic hydroxyapatite (HA) using plasma spray has also been introduced in an attempt to provide early as well as long-term fixation [36]. However, uniform coating of HA in the rough surfaces of the prosthesis is difficult. Our tissue engineering approach shows the appearance of bone forming osteoblasts as well as bone matrix on the surface of CoCr implants. The approach needs seeding of mesenchymal cells on the rough surface of the implants. As the cells are suspended in a culture medium, they can easily inhabit even deep surface areas. Therefore, the mesenchymal cells are distributed uniformly, and further differentiate into osteoblasts which fabricate bone matrix. Importantly, the matrix contains low crystallized carbonate containing HA, therefore so called biological HA exist in the tissue engineered implants [9]. Biological HA is known to show greater biocompatibility compared to synthetic HA. These facts indicate that our approach enabled us to coat the implant surface uniformly with a viable HA layer. Moreover, active osteoblasts with osteogenic function are present in the implant surface. Thus, our method is unique with regard to coating HA concomitant with active osteoblasts on the CoCr alloy implant surface. That is to say, we can generate an HA coating layer with osteogenic function.

In this paper, we described the tissue engineering approach using mesenchymal cells on metallic implants intended for orthopedic applications; however we believe this approach might also be appropriate for other applications such as maxillofacial prosthesis. Further studies are needed to provide evidence of suitability in other clinical applications.

## 3. Materials and Methods

### 3.1. Preparation of Marrow Mesenchymal Cells and Implants

Approval from the animal experimental review board of Nara Medical University was obtained prior to the start of the study. Mature male Japanese white rabbits weighing about 3.0 kg were used. Six rabbits were anesthetized and 2 mL of bone marrow was aspirated from the humerus of each rabbit. The bone marrow aspirates were placed in a T-75 flask (Coster Co., Cambridge, MA) and mixed with 15 mL of Eagle mimimum essential medium (MEM; Nacalai Tesque, Inc., Kyoto, Japan) containing 15% fetal bovine serum (FBS; JRH Biosciences, Lenexa, KS) and a mixture of antibiotics (100 Unit/mL penicillin, 100 mg/mL streptomycin, and 0.25 mg/mL amphotericin B; Sigma Chemicals Co., St. Louis, MO). The primary cultures were maintained in a 5% CO_2_ atmosphere at 37 °C. After 2 days of culture, non-adherent cells were removed and 20 mL of the culture medium was added. The following medium change was done at 3 times per week. Floating cells (red blood cells and hematopoietic cells) were removed during these medium changes. After 2 weeks of the primary culture, the number of adherent cells with fibroblastic shape increased. The adherent cells were termed mesenchymal cells in the present study.

The cultured mesenchymal cells were then released with 0.25% trypsin, centrifuged at 900 rpm for 5 min at room temperature and condensed at a cell density of 1 × 10^6^ cells/mL. Cell number was counted by CDA-1000 (Sysmex, Kobe, Japan). Two milliliter of the cell suspension (2 × 10^6^ cells) was applied on one side of an implant surface and incubated overnight, then subcultured. The subcultures were done on six-well plates for 2 weeks with MEM containing 15% FBS, 10^−8^ M dexamethasone, 10 mM β-glycerophosphate, and 0.07 mM ascorbic acid. This allowed differentiation of the mesenchymal cells to osteoblasts and formation of bone matrix on the implant surface [7,9–11].

**Figure 5:**
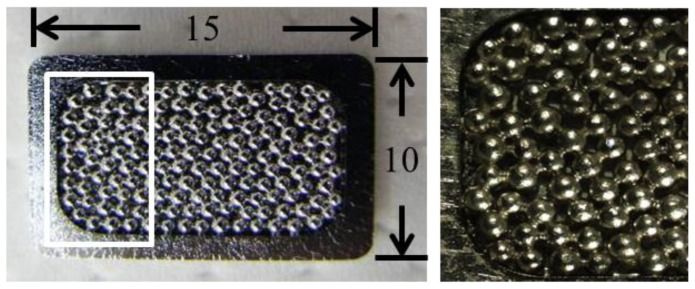
Cobalt-Chromium (CoCr) based alloy. The surfaces were coated with a single layer of 710 to 850 μm diameter CoCr based alloy beads. Right figure is a magnified image of the rectangular area in the left figure. The size of the alloy is 15 × 10 × 2 mm.

The implants used in the present study were Cobalt-Chromium (CoCr) based alloy (Japan Medical Materials Co., Osaka, Japan) measuring 15 × 10 × 2 mm. The surfaces were coated with a single layer of 710 to 850 μm diameter CoCr based alloy beads (Figure 5).

### 3.2. Implantation

**Figure 6:**
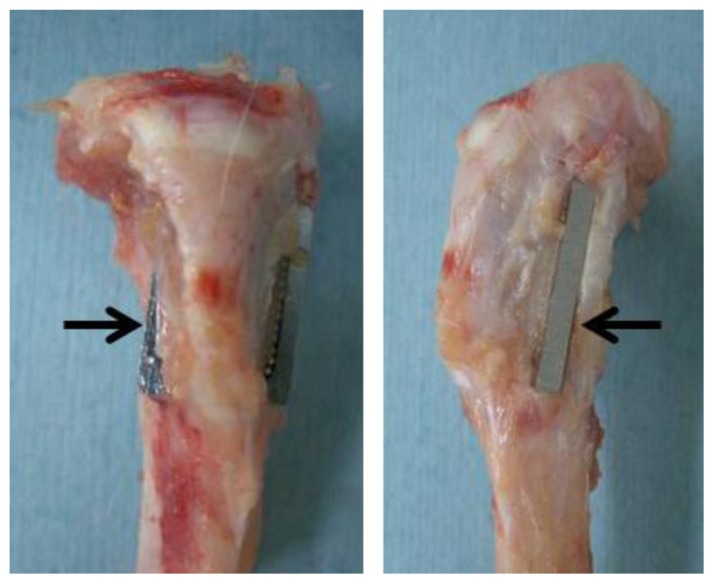
Implantation of the CoCr alloy in rabbit tibial bone defect. Left and right figure are anterior-posterior and lateral view, respectively. Arrows indicate the implant which inserted into the bone defect.

After the subculturing, the CoCr alloys were implanted under general anesthesia bilaterally in the tibias of the rabbits from which bone marrow had been aspirated; therefore we used autogeneic marrow mesenchymal cells. Using sterile surgical technique, a 2-cm longitudinal skin incision was made on the anteromedial aspect of the proximal metaphysis of the tibia. The fascia and periosteum were incised and retracted to expose the tibial cortex. Using a 2-mm electric steel dental burr, a 15 × 10 × 2 mm opening was made from the medial cortex to the lateral cortex parallel to the longitudinal axis of the tibial metaphysis. After irrigating the opening with saline, the CoCr alloy was implanted in the frontal direction, perforating the tibia, and protruding from the medial-to-lateral cortex symmetrically with respect to the longitudinal axis of the tibial metaphysis [37,38]. The cell loaded side was implanted facing the anterior surface of the tibia in the right leg, and facing the posterior surface in the left leg. The interface between the implant and bone was not the same on both sides due to the triangular cross-sectional geometry of the tibia; the anterior side had a smaller facing area than the posterior side. Therefore the CoCr alloys were implanted in opposite directions in the right and left tibias to offset differences in test sites (Figure 6). Three weeks after implantation, each rabbit was sacrificed, and the tibias with implants were extracted to evaluate the pull-out force in the mechanical test.

### 3.3. Alizarin Red S Staining

For the Alizarin Red S staining, the subcultured cell layers were washed twice with PBS (−) (phosphate-buffered saline without Ca^2+^ and Mg^2+^). After fixing with 95% ethanol (4 °C, 15 min), they were washed with deionized water and then 0.4 mL of Alizarin Red S (Nacalai Tesque Inc.) solution dissolved in the PBS (5 mg/mL) was added to the culture well. After 1 min, the wells were washed several times with deionized water to remove the remaining stain.

### 3.4. Measurement of Pull-out Force

**Figure 7:**
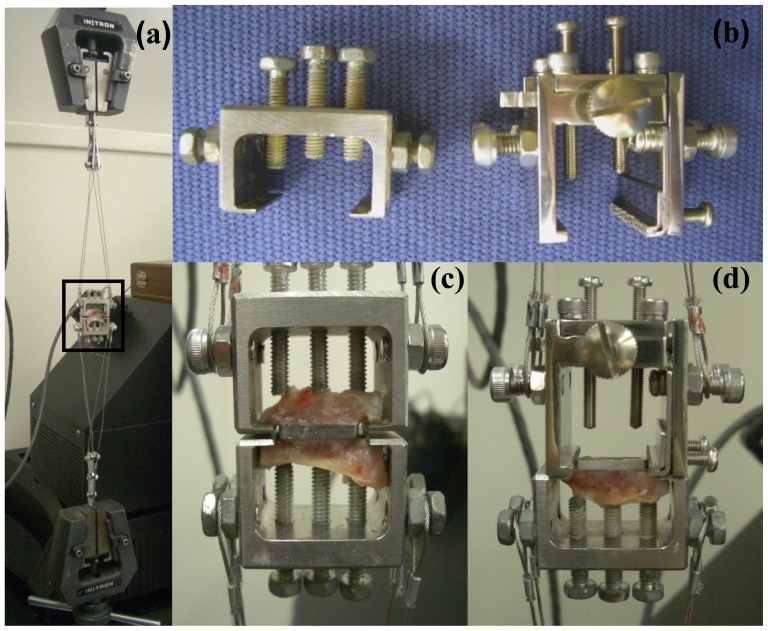
Mechanical test (pull-out force). (**a**) Whole image of the mechanical test; (**b**) Left photo shows a grip for first pull-out test and right shows a grip for the second pull-out test. The grip for the second test was used to grasp the implant directly; (**c**) Image of first pull-out test. This is a magnified image of the rectangular area in Figure a; (**d**) Image of the second pull-out test.

Eight of the 12 limbs with implants were examined by mechanical test. Two implants were excluded because of loosening due to infection in one limb. Thus, tibias from 6 limbs were used for this test. The specimens were trimmed with the implants sandwiched above and below the tibia, and were prepared for the detachment test using the method of Nakamura [37]. These specimens were positioned horizontally, and the upper and lower bone cortex was placed in a special grip. Mechanical pull-out force was evaluated under tension at a crosshead speed of 0.5 mm/min (Figure 7). First, there was detachment of the implant from the side with weaker bonding. Maximum pull-out force was measured at detachment of the implant from bone. After the first pull-out test, a second pull-out test between the CoCr implant which was held directly with another grip and remaining cortex bone on other side was performed.

### 3.5. Histological Examination and Electron Microscope Analysis

Four of the 12 limbs with implants were examined by histological examination and electron microscope analysis. These specimens were fixed with 10% neutral buffered formalin**,** and then embedded in polyester resin (Ohken, Tokyo, Japan). The resin-embedded tissue blocks were sectioned into midportion perpendicularly to the long axis of the implants using a sawing machine (BS3000N, Exakt, Norderstedt, Germany), and ground to final thickness of about 100 μm using a microgrinding machine (MG4000, Exakt, Norderstedt, Germany). The undecalcified sections were then stained with toluidine blue [11].

Each block was sectioned at the implantation site and the surface of the section was polished using waterproof-paper. After setting polished sections in the chamber, sections were examined using a scanning electron microscope (SEM, S-3400N, HITACHI, Japan) and SEM images were acquired.

### 3.6. Statistical Analysis

Pull-out forces for the cell-loaded and noncell-loaded sides of each implant were compared using a Wilcoxon test. Differences with a *p*-value of <0.05 were considered statistically significant.

## 4. Conclusions

We have succeeded in differentiating the mesenchymal cells into active osteoblasts concomitant with bone matrix formation on the CoCr based alloy implant surface using rabbit bone marrow culture. The culture of the mesenchymal cells on the CoCr alloy implant surface prior to implantation resulted in a stable interface between the implant surface and host bone just 3 weeks after implantation. The present findings indicate early fixation of CoCr based alloy by our tissue engineering approach, which might lead to the desired solution of cementless fixation in various joint arthroplasties using CoCr alloy implants.
